# The Assembly of DNA Amphiphiles at Liquid Crystal-Aqueous Interface

**DOI:** 10.3390/nano6120229

**Published:** 2016-12-01

**Authors:** Jingsheng Zhou, Yuanchen Dong, Yiyang Zhang, Dongsheng Liu, Zhongqiang Yang

**Affiliations:** Key Laboratory of Organic Optoelectronics & Molecular Engineering of the Ministry of Education, Department of Chemistry, Tsinghua University, Beijing 100084, China; zhoujs2003@sina.com (J.Z.); dongyuanchen_01@126.com (Y.D.); zhangyiyang13@mails.tsinghua.edu.cn (Y.Z.); liudongsheng@tsinghua.edu.cn (D.L.)

**Keywords:** liquid crystal, interface, self-assembly, DNA amphiphiles, detection

## Abstract

In this article, we synthesized a type of DNA amphiphiles (called DNA-lipids) and systematically studied its assembly behavior at the liquid crystal (LC)—aqueous interface. It turned out that the pure DNA-lipids at various concentrations cannot trigger the optical transition of liquid crystals from planar anchoring to homeotropic anchoring at the liquid crystal—aqueous interface. The co-assembly of DNA-lipid and l-dilauroyl phosphatidylcholine (l-DLPC) indicated that the DLPC assembled all over the LC-aqueous interface, and DNA-lipids prefer to couple with LC in certain areas, particularly in polarized and fluorescent image, forming micron sized net-like structures. The addition of DNA complementary to DNA-lipids forming double stranded DNA-lipids caused de-assembly of DNA-lipids from LC-aqueous interface, resulting in the disappearance of net-like structures, which can be visualized through polarized microscope. The optical changes combined with DNA unique designable property and specific interaction with wide range of target molecules, the DNA-lipids decorated LC-aqueous interface would provide a new platform for biological sensing and diagnosis.

## 1. Introduction

Liquid crystal (LC) is a type of physical form between solid and liquid. It is well recognized for display applications until two decades ago that LCs can be used for sensing and detection. It is attributed to two important properties of LCs, such as the amplification of ordering perturbation of one LC molecule to its adjacent thousands molecules, and the resultant molecular ordering change can be transduced to observable optical signal. It has reported that the orientational transitions of LC materials can reflect interactions of many biological matters [1,2], such as lipid, protein, nucleic acid, virus, bacteria, mammalian cell, etc. [3,4,5,6,7,8,9,10,11,12,13,14,15,16,17,18,19,20,21,22,23]. These objects usually need fluorescent labelling or complex instrumentation through conventional methods. However, LC materials demonstrate its unique advantage to monitor target molecules directly with polarized microscope [24]. So far amphiphilic molecules have been well studied for their assembly behavior at the LC-aqueous interface. The hydrophobic tails penetrate into hydrophobic LCs, and the hydrophilic heads expose to the aqueous solution. These amphiphilic molecules are typically surfactants, lipids and polymers, therefore, the LC-aqueous interface decorated with those amphiphiles usually lacks of specific interactions with target molecules. To address this problem, DNA stands out among other materials, it can be specifically programmed, synthesized and more importantly, the DNA aptamer form can specifically interacts with its target molecules, thus expanding LC-aqueous interface for wide range of detections. From 2008, Schwartz group [25,26,27,28] has explored to add DNA onto LC-aqueous interface, it mainly relies on the non-specific electrostatic interaction between negative DNA and positive surfactant. Later Kun-Lin Yang group [29] directly assembled DNA-cholesterol onto LC-aqueous interface. However, different DNA-amphiphiles may behave differently and here we explore DNA-lipids and systematically study the assembly behavior at the LC-aqueous interface.

## 2. Results and Discussion

**Figure 1:**
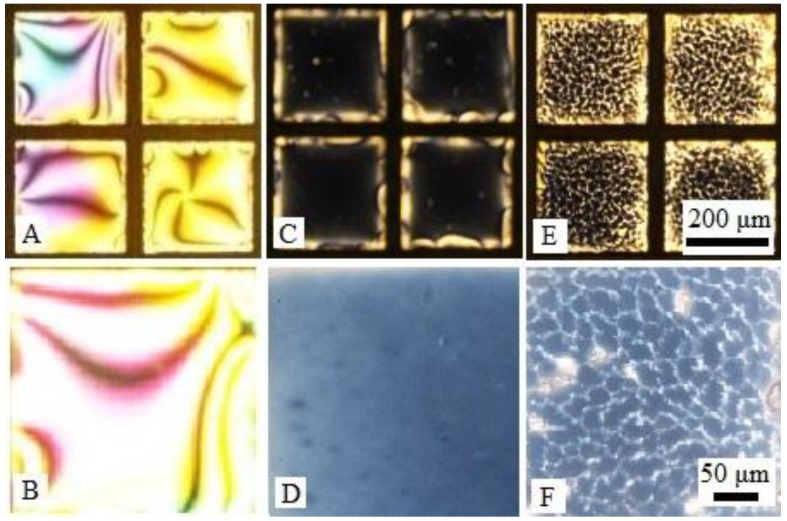
(**A**) Optical images (transmission through crossed polars) of 5CB before and (**B**) after exposure to aqueous (phosphate buffer saline (PBS)) dispersions of 20 μM DNA-lipids for 1 h (magnified image); (**C**) Optical images of 5CB exposure to aqueous dispersions of 5 μM l-DLPC for 1 h and (**D**) magnified image; (**E**) Optical images of 5CB exposure to aqueous dispersions of 20 μM DNA-lipids and 5 μM l-DLPC for 1 h and (**F**) magnified image.

We first synthesized DNA-lipids, which contain two 18-carbon chains and a negative hydrophilic 20-nt DNA as polar head group. We employed solid-phase synthesis, the 1,2-*O*-Dioctadecyl-rac-glycerol first reacted with 2-cyanoethyl-*N*,*N*-diisopropylphosphoramidite, and then attached it to DNA 5′ terminals. The DNA sequence is 5′-CGA TGC TCA GTA CGA GGA CT-3′. Both matrix-assisted laser desorption/ionization time of flight (MALDI-TOF) mass spectroscopy (Figure S1) and polyacrylamide gel electrophoresis (Figure S2) verified that the DNA-lipids were indeed obtained as designed. Next, we studied the assembly behavior of DNA-lipids at the LC-aqueous interface. In a typical experiment, nematic 4-cyano-4′-pentylbiphenyl (5CB) was used and supported on a hydrophobic *N*,*N*-dimethyl-n-octadecyl-3-aminopropyltrimethoxysilyl chloride (DMOAP)-treated glass. The LC film was about 20 μm thick, confined by a gold grid through capillary force. In order to spare the use of DNA-lipid, we exploited a silicone isolator, which allows to isolate specimens using removable hydrophobic barriers. 25 μL PBS buffer was slowly added onto 5CB film by a pipette tip. Once 5CB contacted with water, it appeared bright optical appearance under crossed-polars, attributing to the parallel alignment of 5CB to the aqueous interface, see Figure 1A. After adding a stated amount of DNA-lipid buffer solution, the sample was examined under the polarized microscope. Figure 1B showed that various DNA-lipid concentrations ranging from 2 to 100 μM (Figure S3) did not induce a visible optical change, indicating that 5CB did not undergo a parallel to perpendicular transition of 5CB. Pure DNA-lipid did not cause an optical transition as conventional amphiphilic molecules do, it might be due to following reasons: (i) the size of hydrophilic DNA is too big to be accommodated at the LC-aqueous interface closely; (ii) the negative charges of DNA cause strong repulsion; (iii) the assemblies of DNA-lipids are too stable to disassemble, therefore not able to assemble at the LC-aqueous interface; (iv) the DNA chain has non-specific interactions with 5CB. Considering all above factors, we conducted co-assembly of DNA-lipids with l-DLPC so that l-DLPC can penetrate between DNA-lipids and promote the assemblies of DNA-lipids to disassemble, leading free DNA-lipids to assemble at the LC-aqueous interface. The presence of l-DLPC can also lower the repulsion between DNA-lipids and decrease the non-specific interaction between DNA and 5CB interface. We first carried out control experiment, assembling 5 μM l-DLPC at the LC-aqueous interface, as shown in Figure 1C and 1D, the bright optical image turned to dark, due to the transition of 5CB from parallel to homeotropic alignment caused by l-DLPC. In contrast, after addition of 20 μM DNA-lipids and 5 μM l-DLPC for 1 h, the optical image of 5CB turned to net-like structure (Figure 1E,F). This new optical change has not been reported previously, and is different from the optical image of solely DNA-lipids or l-DLPC caused, nor the mixture of DNA and l-DLPC (Figure S4).

**Figure 2:**
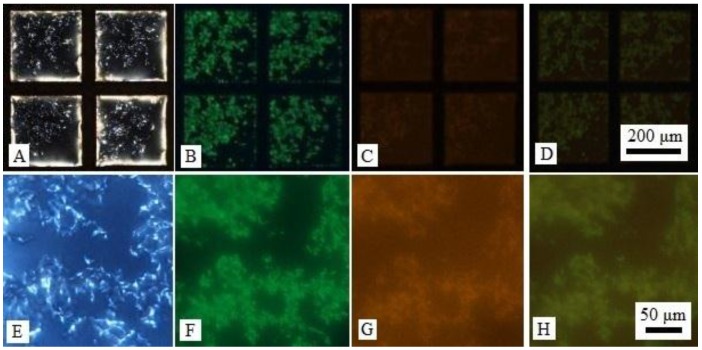
(**A**) Optical images (crossed polars) and (**B**) corresponding green fluorescence micrographs and (**C**) corresponding red fluorescence micrographs of LC-aqueous interface exposure to a mixture of 20 μM DNA-lipids, 5 μM l-DLPC and 0.5 μM Rh-DHPE for 1 h then introduction of 1× SYBR Green; (**D**) Overlaid images of (**B**,**C**); (**E**–**G**) Magnified image of (**A**–**C**); (**H**) Overlaid images of (**F**,**G**).

For more understanding the LC-aqueous interface decorated with mixture of DNA-lipids and l-DLPC, we used l-DLPC doped with 10 mol % fluorescently labeled lipid Rhodamine B 1,2-dihexadecanoyl-sn-glycero-3-phosphoethanolamine triethylammonium salt (Rh-DHPE) to indicate the location of l-DLPC at interface, and fluorescent dye SYBR Green binding with DNA strands to indicate the location of DNA-lipids. In a typical experiment, LC-aqueous interface was incubated with 50 μL solution containing 20 μM DNA-lipids, 5 μM l-DLPC and 0.5 μM Rh-DHPE for 1 h, and the sample was washed three times by replacing with PBS buffer while keeping the interface from exposing to air. It was observed that the LC-aqueous interface appeared net-like structures as shown in Figure 2A and magnified image in Figure 2E. The corresponding fluorescent image indicated that the net-like structure are the regions of DNA-lipids rich region. However, the Figure 2C,G indicated that the l-DLPC assembled across the entire LC-aqueous interface. It is worth mentioning that the optical image of LC-aqueous interface slightly different from the net-like structures shown in Figure 1E,F when Rh-DHPE existed, it assumed more complex structure formed due to the presence of Rh-DHPE, but it did not affect our discussion about assembly position of the mixture at the LC-aqueous interface.

We also explored how different ratios of DNA-lipids and l-DLPC influence the assembly behavior at the LC-aqueous interface. The total concentration of DNA-lipids and l-DLPC was kept at 10 μM, and the ratio of DNA-lipids and l-DLPC varied from 7.5:2.5 to 5:5 (Figure S5). The fluorescent experiments were conducted and it was observed that the bright domains with high fluorescence intensity was corresponding to the domains enrichment of DNA-lipids (Figure S6). The more DNA-lipids, the wider and bigger of net-like structures. We also conducted a fluorescent experiment that proved 20-nt ssDNA at the concentration of 20 μM did not cause detectable adsorption of DNA onto the LC-aqueous interface (Figure S7). Therefore, the non-specific interaction between DNA and 5CB was very low and the net-like structures are likely formed due to the co-effect of DNA-lipids and l-DLPC. Due to electrostatic interaction of DNA with l-DLPC, the local LC alignment was disrupted and appeared to be bright net-like structures.

**Figure 3:**
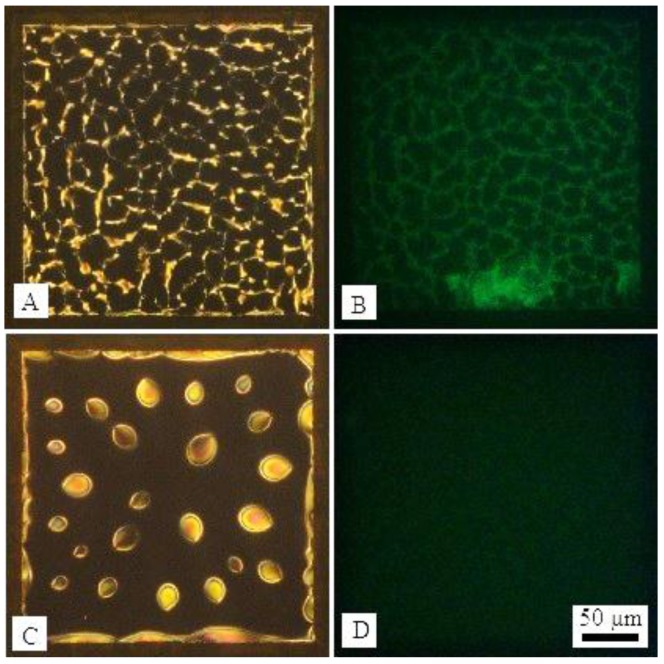
(**A**) Optical images (crossed polars) and (**B**) corresponding fluorescence micrographs of LC-aqueous interface decorated with DNA-lipids and l-DLPC, and dyed by SYBR Green; (**C**) Optical images and (**D**) corresponding fluorescence micrographs after the introduction of 20 μM c-DNA of DNA-lipids for 3 min in (**A**).

**Figure 4:**
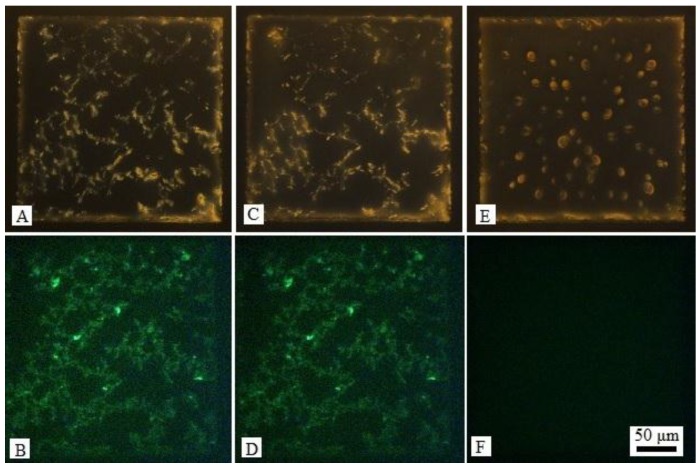
(**A**) Optical images (crossed polars) and (**B**) corresponding fluorescence micrographs of LC-aqueous interface decorated with DNA-lipids and l-DLPC, and dyed by SYBR Green; (**C**) Optical images and (**D**) corresponding fluorescence micrographs after the introduction of 20 μM random DNA strands for 10 min in (**A**); (**E**) Optical images and (**F**) corresponding fluorescence micrographs after the introduction of 20 μM c-DNA of DNA-lipids for 3 min in (**C**).

The assembled locations of DNA-lipids and l-DLPC have been clarified, we next explored whether the LC-aqueous interface can respond to DNA strands. The LC-aqueous interface was first decorated with DNA-lipids and l-DLPC (Figure 3A) by exposing 20 μM DNA-lipids, 5 μM l-DLPC as described before, after 1 h incubation, the sample was washed to remove free amphiphiles. Then complementary DNA strands (c-DNA) to DNA-lipids was added, resulting in a final concentration of 20 μM. It was observed that the optical image of LC changed obviously, particularly the net-like structures disappeared and the image turned to dark with bright ellipsoidal domains in 3 min, Figure 3A,C. This is a very typical pattern of l-DLPC assembled at the LC-aqueous interface at the early stage [30]. By using SYBR Green that can bind with DNA strands as fluorescence label, we determined that the net-like structures corresponding to green fluorescence domains, Figure 3B. In contrast, after the addition of c-DNA, the fluorescent net-like structures disappeared, and the fluorescence intensity diminished across the entire LC-aqueous interface. It is likely because the c-DNA hybridized with DNA-lipids, resulting in the desorption of DNA-lipids from LC-aqueous interface and release of DNA-lipids into aqueous solution, Figure 3D. Note that the l-DLPC leftover re-assembled and formed ellipsoidal domains as lipids usually do, further confirming the escape of DNA-lipids from the LC-aqueous interface. The control experiment adding non-complementary 20-nt ssDNA strands such as random DNA sequence, showed that the optical image of LC did not change, Figure 4. Again, once the complementary DNA strands were added, DNA-lipids were driven away from the interface through hybridization, likely the hybridized DNA-lipids with DNA double strands as the hydrophilic groups whose size was doubled comparing to single stranded DNA. The interaction between hydrophobic carbon tails of DNA-lipids and LC is not strong enough to hold the increased bulky hydrophilic groups, leading to the disappearance of net-like structures.

**Figure 5:**
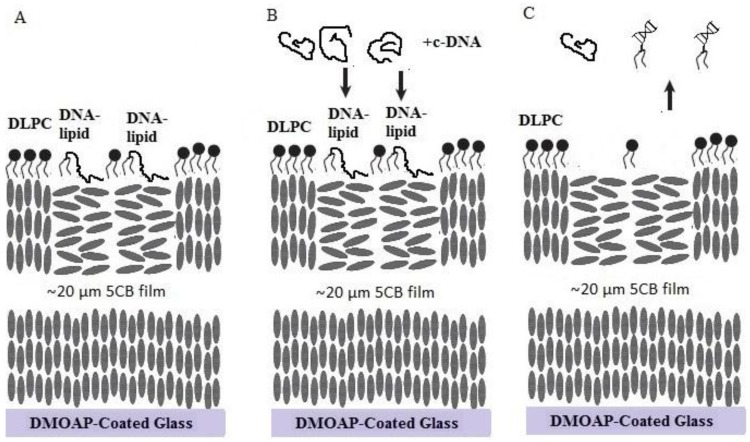
The schematic illustration of the LC-aqueous interface decorated with DNA-lipids and l-DLPC, (**A**) before and (**B**) after addition of its complementary DNA strands, c-DNA. (**C**) DNA-lipids hybridize with c-DNA to form double strands and disassemble from the LC-aqueous interface.

Combined all the observations, we illustrate a scheme to describe the interfacial phenomenon of how LC-aqueous interface decorated with DNA-lipids and l-DLPC interacts with its complementary DNA. As shown in Figure 5. First, DNA-lipids and l-DLPC assembled at LC-aqueous interface, with DNA-lipids rich region located at the net-like structures and l-DLPC at the entire surface. After adding the complementary ssDNA strands, DNA-lipids hybridized to form double strands and disassembled from the LC-aqueous interface leaving l-DLPC behind, consequently, the optical image changed from net-like structures to dark with bright ellipsoidal shape domains.

**Figure 6:**
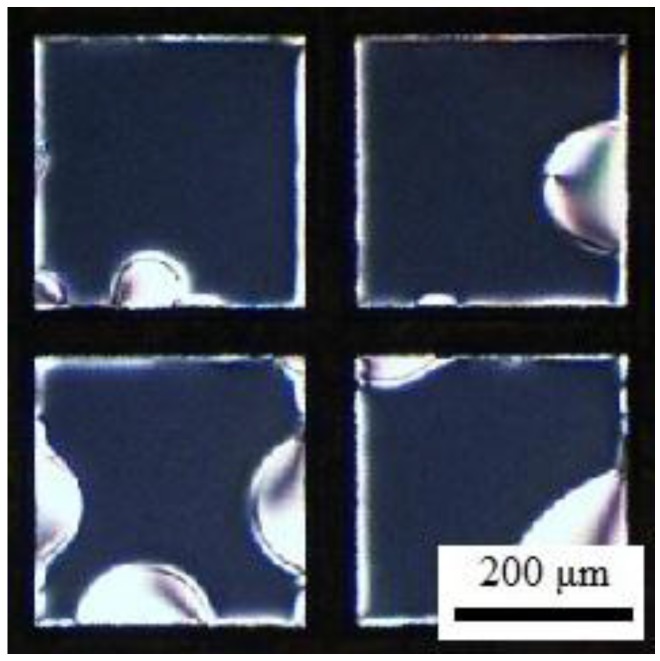
Optical images (crossed polars) of 5CB exposure to a mixture of 20 μM DNA-lipids, 20 μM c-DNA and 5 μM l-DLPC for 1 h.

We make a final observation regarding to the assembly of DNA-lipids and l-DLPC at LC-aqueous interface. Different from previous experiment shown in Figure 2 and Figure 3, the DNA-lipids and l-DLPC were mixed with complementary DNA strands before contacting with LC-aqueous interface. As illustrated in Figure 6, net-like structures were not observed at the LC-aqueous interface, indicating that the DNA-lipids did not assemble at the interface. In addition, the bright micron size domains are typical patterns during the assembly of l-DLPC at the LC-aqueous interface. This provides further evidence that once DNA-lipids hybridize to double stranded form, they prefer to stay in aqueous solution instead of assembling at the LC-aqueous interface.

## 3. Materials and Methods

### 3.1. Materials

DMOAP, 5CB was purchased from Aldrich. 1,2-*O*-Dioctadecyl-rac-glycerol was purchased from Santa Cruz Biotechnology, Inc. (Shanghai, China). *N*,*N*-Diisopropylethylamine (DIPEA) was purchased from Alfa Aesar (Shanghai, China). 2-Cyanoethyl *N*,*N*-diisopropylchlorophosphoramidite was purchased from Alfa Aesar (Tianjing, China). 5-(Ethylthio)-1H-tetrazole (ETT) was purchased from GenePharma Co. Ltd (Shanghai, China). Rh-DHPE was purchased from Invitrogen (Shanghai, China). SYBR Green was purchased from Biomics Biotechnologies Co. Ltd (Nantong, China). l-DLPC was purchased from Avanti (Alabaster, AL, USA). Other salts and solvents were purchased from Tianjing FUCHEN chemical reagents factory (Tianjin, China). Water used in all experiments was Milli-Q (Billerica, MA, USA) deionized (18.2 MΩ·cm^−1^). Silicone isolators were purchased from Grace Bio-Labs (Bend, OR, USA). Gold grids were purchased from Electron Microscopy Sciences (Hatfield, PA, USA). Glass slides were purchased from Fisher Scientific (Waltham, MA, USA).

The DNA sequence of DNA-lipids: HO-5′-CGA TGC TCA GTA CGA GGA CT-3′.

The c-DNA sequence: 5′-AGT CCT CGT ACT GAG CAT CG-3′.

The random sequence: 5′-AGT AGT GGA CCG ATA GAT GA-3′.

### 3.2. Synthesis of DNA-Lipids

The DNA-lipids is synthesized by previous reported solid-synthetic method [31].

1,2-*O*-Dioctadecyl-rac-glycerol (0.6 g, 1 mmol) was dissolved in anhydrous tetrahydrofuran (THF) (10 mL). *N*,*N*-Diisopropylethylamine (DIPEA, 0.65 g, 5.0 mmol, 0.90 mL) was added followed by dropwise addition of 2-cyanoethyl *N*,*N*-diisopropylchlorophosphoramidite (0.32 g, 1.35 mmol, 0.30 mL). After 30 min, the reaction mixture was diluted with ethyl acetate (50 mL). The solution was washed with water (50 mL) for 3 times and saturated aqueous solution of NaCl (50 mL), dried by anhydrous Na_2_SO_4_ and concentrated. After purified by flash column chromatography, compound **1** (Figure S8) was obtained as little yellow oil.

The controlled pore glass (CPG) loaded DNA was synthesized using ABI 394 DNA synthesizer in 1 μmol scale with a standard DNA synthesis protocol. The DNA-loaded CPG T_1_ (4 μmol) was transferred into a vial, then compound 2 (0.32 g, 0.4 mmol) and ETT (0.1 g, 0.8 mmol) were added consequently. After dried in vacuum, 5 mL anhydrous THF was added under dry nitrogen protection. The reaction mixture was allowed to stay overnight under room temperature. Then CPG was washed twice with anhydrous THF followed by treated with iodine and water in THF. After cleaved by concentrated ammonia solution in 60 °C for 3 h, the crude product was purified by High Performance Liquid Chromatography (HPLC). The product was purified by HPLC with the acetonitrile (ACN) content increasing from 50% to 90% in 10 min and then keeping 90% ACN for 5 min. The final product was detected by MALDI-TOF: MALDI-TOF calcd for DNA-lipids 6799, found 6796.

### 3.3. Preparation of DMOAP-Treated Glass

Glass slides were cleaned with piranha solution. Briefly, the slides were treated by piranha solution (70% (*v*/*v*) sulfuric acid and 30% (*v*/*v*) hydrogen peroxide) for one hour at approximately 80 °C. The slides were then rinsed with about 3 L water. After it, the slides were immersed in 1.5 vol % DMOAP solution for 1 h at room temperature. They were then rinsed with water for 3 times and methanol for 2 times and dried under nitrogen. The quality of the DMOAP layer was tested by forming an optical cell. 5CB was dropped into the gold grid on the slides and the resulting optical texture was examined using polarized light to confirm if 5CB was assuming homeotropic anchoring.

### 3.4. Preparation of Optical Cells

The gold specimen grids were cleaned in water and methanol alternately for 3 times and then heated at 90 °C for 1 h. The grids were then placed onto the surface of DMOAP-treated glass slides. 1 μL of 5CB was dispensed onto each grid and the excess LC was removed by a 10 μL syringe. A slice of silicone isolator with holes was put on the glass slide and made sure each grid was approximately at the center of holes. The silicone isolator was pressed to cling to glass slide so that no liquid leaking between the silicone isolator and glass slide. 25 μL PBS buffer was put on the top of 5CB in the hole. Then 25 μL sample solution (DNA-lipids, l-DLPC or mixture with a stated concentration) was dropped into the hole.

### 3.5. Using of SYBR Green to Label DNA Strands

The concentration of SYBR Green product was 10,000×. It was diluted to 100× for using. When the assembly of mixture completed, exchanging the solution with PBS, then 0.5 μL 100× SYBR Green was dropped into 50 μL sample solution (1×). 5 min later, the solution was exchanged with PBS to remove excessive SYBR Green in solution.

### 3.6. The Preparation of l-DLPC and DNA-Lipids Mixture

After mixed, DNA-lipids and l-DLPC were shaked by vortex for about 2 min and then disposed by sonicleaning (40 kHz, 100 W) for about 3 min to make sure that DNA-lipids and l-DLPC can disassemble and assemble at LC-aqueous interface together.

## 4. Conclusions

In conclusion, we report the assembly behavior of DNA-lipids at the LC-aqueous interface. It was found that DNA-lipids can assemble at LC-aqueous interface with l-DLPC, resulting in net-like structures in micron scale. The fluorescent experiments indicated that those net-like structures correspond to DNA-lipids regions whereas l-DLPC assembles across the entire LC-aqueous interface. DNA-lipids and l-DLPC decorated LC-aqueous interface could interact with complementary DNA and DNA-lipids desorb from LC-aqueous interface into aqueous while did not interact with non-complementary DNA strands. Considering the powerful function of DNA aptamer, which can specifically interact with species ranging from ions, nucleic acids, proteins, to cells, such DNA modified LC-aqueous interface provides a platform for universal tunable interface for sensing and diagnosis.

## Supplementary Materials

The following are available online at http://www.mdpi.com/2079-4991/6/12/229/s1.
