# Supplementary material for: The Assembly of DNA Amphiphiles at Liquid Crystal-Aqueous Interface
Source: Nanomaterials (Basel). 2016 Dec 1;6(12):229. doi: 10.3390/nano6120229 (PMC5302708; doi:10.3390/nano6120229)
Supplement: Supplementary file 1 [file nanomaterials-06-00229-s001.pdf]

# Supplementary Materials: The Assembly of DNA Amphiphiles at Liquid Crystal-Aqueous Interface

**Jingsheng Zhou, Yuanchen Dong, Yiyang Zhang, Dongsheng Liu and Zhongqiang Yang**

## 1. Characterization of DNA-Lipids

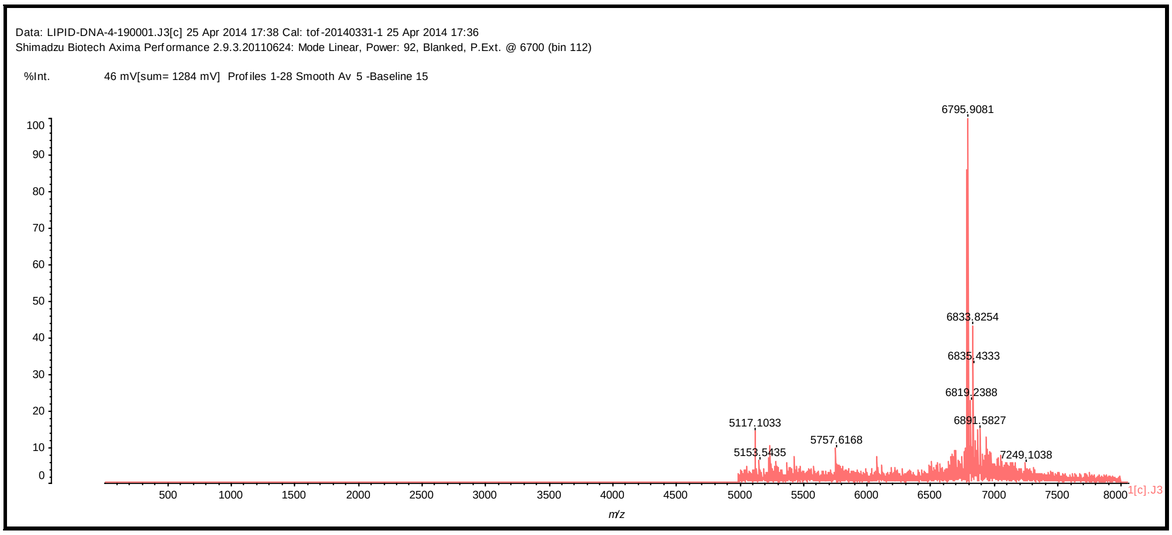


**Figure S1.** MALDI-TOF Spectra of DNA-lipids.

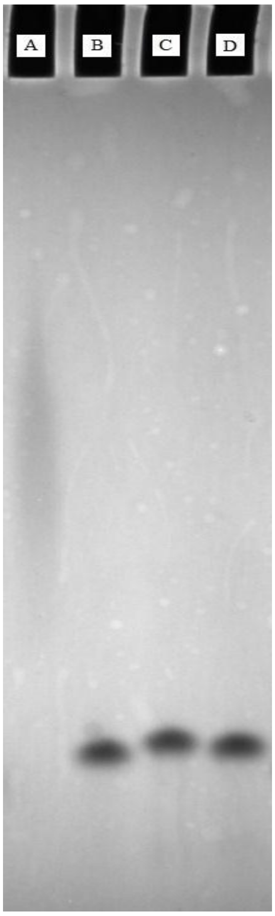


**Figure S2.** Polyacrylamide gel electrophoresis of DNA-lipids and DNA strands. (**A**) DNA-lipids $T_1$; (**B**) c-DNA $T_{1'}$; (**C**) DNA strands $T_1$; (**D**) random strands $T_2$.

## 2. Optical Images (Crossed Polars) of 5CB Contacted with Pure DNA-Lipids at Various Concentrations

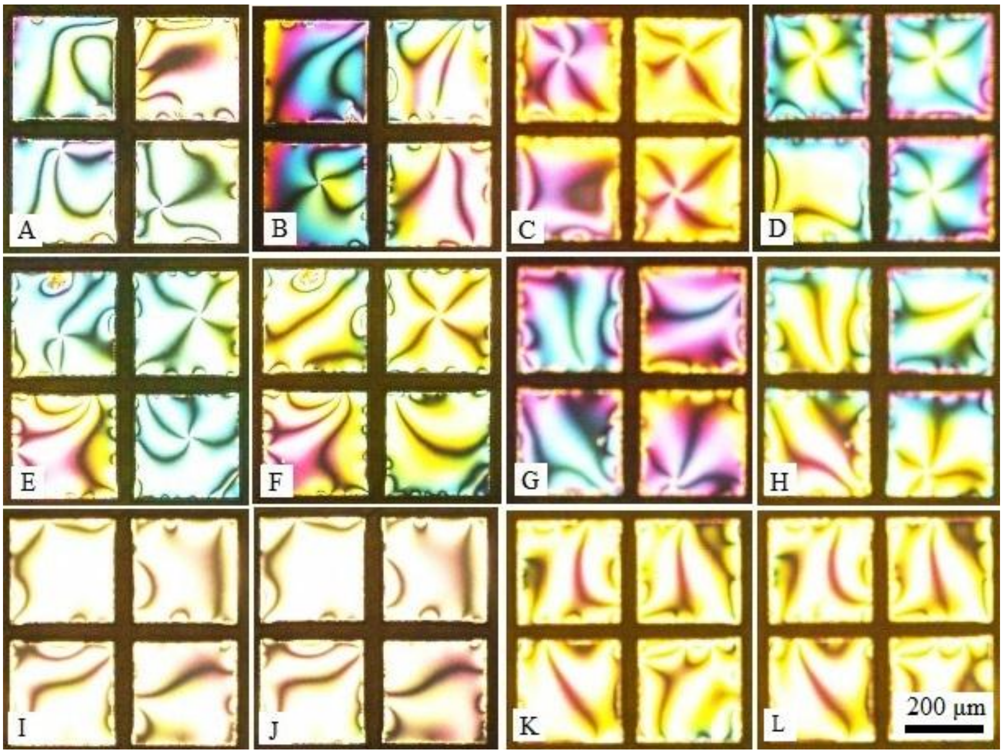


**Figure S3.** Optical images (transmission through crossed polars) of 5CB before (**A**,**C**,**E**,**G**,**I**,**K**) and after (**B**,**D**,**F**,**H**,**J**,**L**) exposure to aqueous (PBS) dispersions of a stated amount of DNA-lipids for 1 hour. The concentration of DNA-lipids: (**A**,**B**) 100 µM; (**C**,**D**) 50 µM; (**E**,**F**) 25 µM; (**G**,**H**) 10 µM; (**I**,**J**) 5 µM; (**K**,**L**) 2 µM.

## 3. Optical Images (Crossed Polars) of 5CB Contacted with a Mixture of DNA and L-DLPC

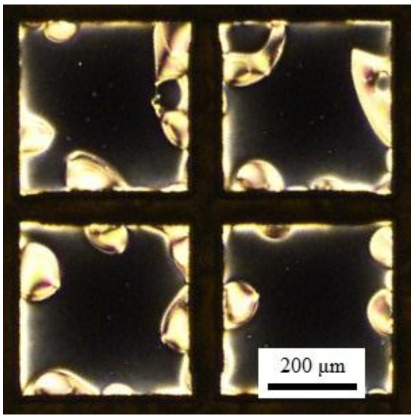


**Figure S4.** Optical images of 5CB exposure to aqueous dispersions of mixture 20 µM DNA and 5 µM L-DLPC for 1 h.

## 4. Optical Images (Crossed Polars) of 5CB Contacted with a Mixture of DNA-Lipids and L-DLPC at Various Ratios

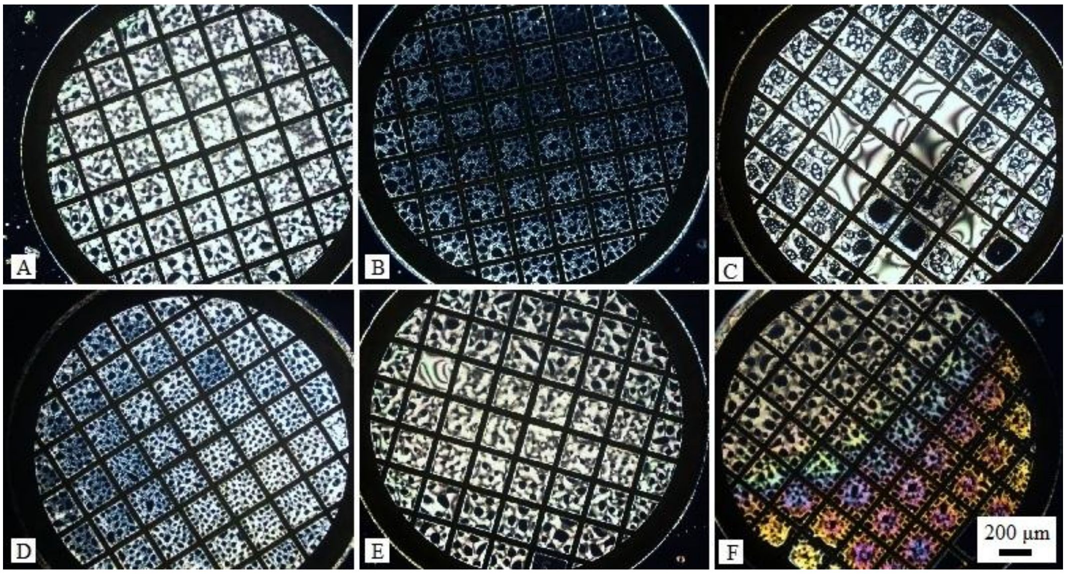


**Figure S5.** (**A**) Optical images (crossed polars) of 5CB exposure to 10 μM mixture DNA-lipids and L-DLPC for 1 h. The concentration of DNA-lipids/L-DLPC: (**A**) 7.5/2.5; (**B**) 7/3; (**C**) 6.5/3.5; (**D**) 6/4; (**E**) 5.5/4.5; (**F**) 5/5.

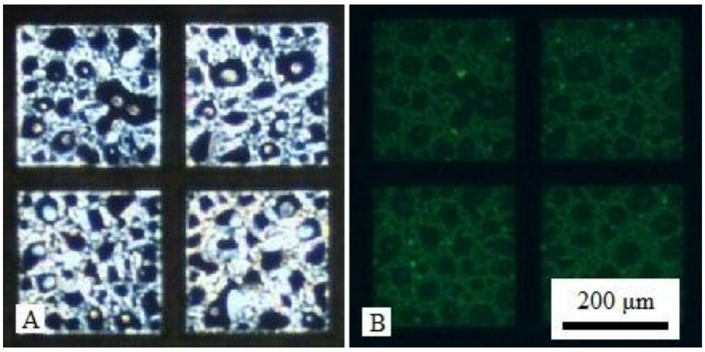


**Figure S6.** (**A**) Optical images (crossed polars) and (**B**) corresponding fluorescence micrographs of 5CB exposure to mixture 6 μM DNA-lipids and 4 μM L-DLPC for 1 h then introduction of 1× SYBR Green.

## 5. Optical Images (Crossed Polars) and Corresponding Green Fluorescence Images of 5CB Contacted with Pure DNA

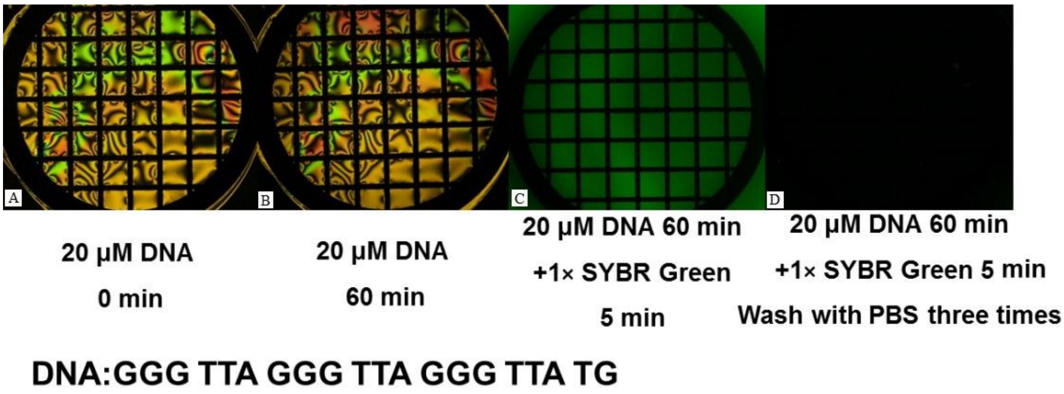


**Figure S7.** Optical images (transmission through crossed polars) of 5CB before (**A**) and after (**B**) exposure to aqueous (PBS) dispersions of 20 μM DNA for 1 h; (**C**) Fluorescent images of 5CB exposure to aqueous dispersions of 20 μM DNA for 1 h and dyed by SYBR Green 5 min, followed by washing with PBS buffer for three times (**D**).

## 6. Synthesis of DNA-Lipids

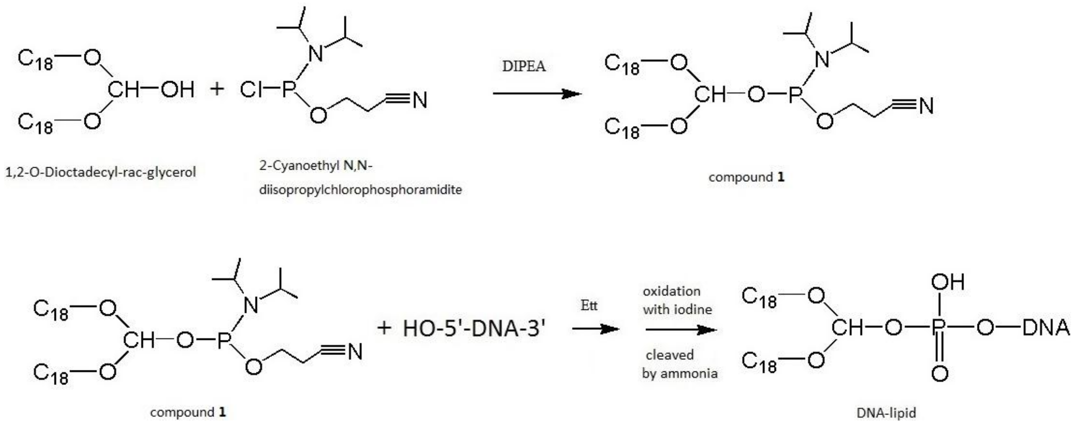


**Figure S8.** The schematic illustration of DNA-lipids synthesis.

© 2016 by the authors; licensee MDPI, Basel, Switzerland. This article is an open access article distributed under the terms and conditions of the Creative Commons by Attribution (CC-BY) license (http://creativecommons.org/licenses/by/4.0/).
